# Low-Cost Qualitative Screening of Melting Ranges in Recycled Fishing Net Plastics: Comparison of Portable Handheld Device with Kofler Hot Bench and DSC

**DOI:** 10.3390/polym18151809

**Published:** 2026-07-24

**Authors:** Sylvia Waschke, Julia Tetzner, Thomas Potempa, Max Ehleben

**Affiliations:** Institute of Recycling (IFR), Ostfalia University of Applied Sciences, 38440 Wolfsburg, Germany; s.waschke@ostfalia.de (S.W.); ju.tetzner@ostfalia.de (J.T.); m.ehleben@ostfalia.de (M.E.)

**Keywords:** plastic recycling, melting range, low-cost portable screening device, Kofler hot bench, differential scanning calorimetry, polyethylene, polypropylene, polyamide, fishing nets, qualitative analysis

## Abstract

Reliable polymer identification is a prerequisite for high-quality recycling of post-consumer fishing nets and ropes. Differential Scanning Calorimetry (DSC) provides precise thermal characterization but requires stationary laboratory infrastructure and is not applicable at coastal collection points or small-scale recycling operations. This study describes and validates Melting Box 1.0 (MB 1.0), a low-cost, field-deployable device for qualitative screening of softening and melting ranges in small polymer samples. MB 1.0 repurposes a commercial vaporizer’s existing temperature-controlled heating and display functionality into a purpose-built screening tool, rather than introducing a new analytical measurement principle. Representative materials from Vietnamese fishing net recycling projects—polyethylene (PE), polypropylene (PP), polyamide (PA), and PE/PP blends—were assessed with a standardized three-tier visual scale (0 = no change; * = softening; x = melting) across five replicate measurements per sample. Agreement with the Kofler hot bench is good (mean bias for T_melt,start_: +2.9 °C; SD = 9.1 °C; *n* = 7), and a systematic offset of +20.6 °C (range +14.1 to +27.6 °C) was observed for PE relative to DSC first-heating peak-end temperatures. The correct material ranking (PE < PP < PA) is preserved, and all four polymer groups are reliably distinguished. At an estimated cost of 150–200 EUR—one to three orders of magnitude below the Kofler hot bench, portable NIR, or DSC—MB 1.0 is intended as a field-deployable screening complement to, not a replacement for, laboratory-grade analytics in resource-constrained recycling contexts.

## 1. Introduction

Reliable separation of thermoplastic polymers by type is a prerequisite for high-quality recycling outcomes. This is particularly relevant for post-consumer fishing nets and ropes, which consist predominantly of polyethylene (PE), polypropylene (PP), and polyamide (PA). Cross-contamination between these polymer classes during collection and sorting reduces recyclate quality and limits the range of viable applications for recovered material [1,2].

A range of analytical methods exist for polymer identification in recycling contexts, spanning laboratory-based and portable approaches. DSC and related thermal techniques (TG and STA) remain the reference standard for quantitative polymer identification and are widely used to characterize plastic marine debris and recycled polyolefin blends [3,4,5]. Spectroscopic methods provide chemical-bond-level identification: ATR-FTIR combined with machine learning has been used to characterize mixed plastic waste [6], and portable or handheld near-infrared (NIR) spectroscopy has been proposed as a field-deployable alternative, including low-cost sensor-based and deep-learning-assisted variants [7,8,9]. Mid-infrared spectroscopy has similarly been explored for chemometric plastic waste discrimination [10]. Recent reviews summarize the broader landscape of plastic waste sorting technologies [11], and low-cost methods for polymer identification in other packaging contexts have also been reported [12]. These methods generally require either stationary laboratory infrastructure or specialized portable instrumentation costing several thousand euros, limiting their applicability at small-scale, resource-constrained collection points. This study arose from field work conducted in the context of the REVFIN project, focusing on the prevention, collection, and recycling of fishing nets from Vietnamese coastal waters [13,14]. A recurring practical challenge was the need to classify polymer types directly at collection points to prevent cross-contamination in the recyclate stream. DSC is the established reference method for quantitative thermal characterization of thermoplastics (ISO 11357-1:2016 [15]) but requires stationary laboratory infrastructure. For sorting operations at collection points, simpler and more mobile solutions are required [16].

Against this background, a portable handheld device (Melting Box 1.0, MB 1.0) was tested for its suitability in qualitative melting range determination of small polymer samples. The device is not conceived as a replacement for laboratory analytics but as a complementary tool for use by operators with limited laboratory training. The aim is to evaluate MB 1.0 systematically by comparison with the Kofler hot bench as an established visual method and DSC as a quantitative laboratory reference. A schematic of the device is provided as Figure S1 (Supplementary Information); the step-by-step operating procedure is illustrated in Figures S2–S10 (Supplementary Information).

## 2. Materials and Methods

### 2.1. Melting Box (MB 1.0)—Device Description

For the experiments on melting behavior of plastics, a portable handheld device (Melting Box 1.0, MB 1.0) was employed. MB 1.0 is a modified commercial vaporizer, not a purpose-built instrument. Its basis is the Flowermate V5.0s PRO dry-herb vaporizer (SMISS Technology Co., Ltd, Shenzhen, China) [17], which already provided the two features essential for this application without modification: a temperature-controlled ceramic heating chamber (40–230 °C in 1 °C increments) and an OLED display for real-time temperature readout ([17], pp. 3–4, 9). For use as a polymer melting-range screening device, the vaporizer’s herb-loading chamber and mouthpiece assembly were replaced with a flat aluminum sample holder machined specifically for this study, and a glass contact rod was added to apply defined pressure to the sample (see Figure S1, Supplementary Information, for the modified layout). All temperature regulation, display, and battery/safety electronics are unmodified original vaporizer components.

The heating element consists of an electrically heated ceramic block (“Advanced Ceramic Heating Chamber”) with a flat-ground surface ([17], pp. 3–4). Temperature is measured by an integrated sensor located within the heating chamber, not in direct contact with the sample, and regulated electronically to defined setpoints; a systematic offset between the displayed sensor temperature and the actual temperature at the sample-contact surface can therefore not be excluded (see Section 2.4). Discrete steps between 130 °C and 230 °C were applied, within the manufacturer-specified range of 40 °C to 230 °C ([17], pp. 3, 9). Temperature is displayed digitally on an OLED display ([17], pp. 3–4, 9).

Samples are placed as small pieces (filaments, flakes, or granulate) into an aluminum tube and positioned on the heating surface. A glass rod applies a defined contact pressure, optimizing thermal contact. Sample masses were kept below 0.5 g to ensure rapid thermal equilibrium and minimize thermal gradients within the specimen; mass was not individually recorded for each run, consistent with the intended field-screening character of the method. A standardized sample weight range could be introduced in future protocol versions to further reduce variability.

Melting behavior is assessed visually using a three-tier scale (illustrated with reference photographs in Figure S9, Supplementary Information):

- 0: no visible change;
*: softening (change in shape, gloss, or tack);
- x: pronounced melting (loss of shape, flow, droplet formation).

The device incorporates several safety features: after 6 min of operation, it enters sleep mode to prevent overheating ([17], pp. 6, 14). The lithium-ion battery (2600 mAh) is charged via an AC/DC adapter; operation during charging must be avoided ([17], pp. 9, 11). Temperature is adjusted via “+” and “−” buttons in 1 °C increments ([17], p. 6).

The MB 1.0 procedure is expressly intended as a qualitative screening method for detecting softening and melting ranges and not as a norm-compliant, high-accuracy melting point determination. A schematic of the device setup is provided as Supplementary Figure S1. The device is operated in accordance with the safety instructions in the original manual ([17], pp. 12–14).

### 2.2. Reference Methods: Kofler Hot Bench and DSC

The Kofler hot bench (Wagner & Munz Type WME) is a stationary instrument with a temperature gradient along a metallic heating surface, established since the 1930s [18]. Samples are moved along the gradient until softening or melting is observed, rated with the same 0/*/x scale. A wooden spatula applies light pressure analogous to the glass plunger rod in MB 1.0, to ensure good thermal contact and minimize differences in contact heat conduction between the two methods.

DSC measurements were performed on selected samples using a NETZSCH DSC 214 Polyma, calibrated with certified standards including indium, following ASTM D7464-18 [19]. Measurements were carried out at a heating rate of 10 K/min under nitrogen atmosphere, using a heat–cool–heat cycle. The reported reference temperature is the end of the melting peak observed during the first heating, rather than the peak temperature of the second heating. This choice reflects the physical state that MB 1.0 and the Kofler hot bench actually assess: the as-received material, with its original processing history, orientation, crystallinity, and any weathering effects intact. The second heating erases this thermal history and is the conventional reference for material identification independent of processing history, but represents a different physical state from the one probed by the field-screening device; using it as the sole comparison would conflate a genuine difference in thermal state between methods with device inaccuracy. First-heating peak-end temperatures, denoted T_peak,end_ DSC for PE, PP, and PA (see Section Transition Temperatures and Comparison with DSC), therefore served as the primary quantitative references.

### 2.3. Samples

Virgin materials, recycled samples, and application-specific specimens were examined, with a focus on fishing nets and ropes from Vietnamese recycling projects. All samples originate from small-scale Vietnamese fishery gear: “virgin” designates new, unused net or rope material, while other samples had been used in fishing operations or, in the case of the polyamide reference (“PA KGU virgin”), were new net material subsequently exposed to prolonged outdoor sun exposure. No manufacturer datasheets were available for this field-sourced material; sample designations reflect the material composition typically documented for Vietnamese small-scale fishery gear (predominantly HDPE nets, minor amounts of polyamide nets, and PE/PP rope blends), not certified polymer grades. Used samples may additionally carry fishery-related surface contamination (biofouling, salt residues, and oils) in addition to manufacturing additives. The dataset was extended beyond the initial measurements to include NTU virgin net, NTU roof 2-month weathered net, PE/PP bright rope (washing series: 0–5 cycles), and PA KGU virgin. An overview is given in Table 1. The complete color-coded temperature matrix for all samples and methods is provided as Figure S11 (Supplementary Information).

### 2.4. Calibration and Derivation of Temperature Parameters

Traceable calibration of MB 1.0 was not possible due to the absence of suitable contact sensors. Spot checks with a surface thermometer confirmed the general plausibility; deviations of several tens of degrees Celsius cannot be excluded. Temperature values from MB 1.0 are treated as nominal values throughout. The Kofler hot bench is calibrated using a reference material at 163 °C; DSC is calibrated with certified standards.

For each sample, five complete temperature runs were performed. Characteristic temperatures derived from the 0/*/x observations:

- T_soft,start_: lowest temperature at which * (softening) was observed in at least one replicate.
- T_melt,start_: lowest temperature at which x (melting) was observed in at least one replicate.
- T_melt,end_: temperature at which x (melting) was observed in all five replicates. Unlike DSC endset temperatures, which denote the physical completion of melting within a single measurement, T_melt,end_ is defined empirically as a replicate-consensus threshold: it identifies the temperature above which melting is consistently observed across independent trials. For field operators using the 0/*/x scale to make sorting decisions, this conservative definition minimizes false negatives (samples wrongly classified as unmelted).

Statistical analysis of the extended dataset confirms the findings of the original dataset: mean absolute deviation MB 1.0 vs. Kofler for *n* = 9 PE sample pairs = 2.8 °C; mean coefficient of variation for DSC triplicates = 0.64%. The washing experiment (0–5 cycles on PE/PP bright rope) to reduce surface contamination shows no statistically significant change in DSC peak temperatures (PE-peak: 141.1–141.8 °C; PP-peak: 176.6–177.5 °C). The complete color-coded temperature matrix is shown in Figure S11 (Supplementary Information).

## 3. Results

### Transition Temperatures and Comparison with DSC

Transition temperatures derived from five replicate measurements on MB 1.0 and the Kofler hot bench are summarized in Table 2. Because both methods rely on discrete temperature steps rather than continuous readout, replicate measurements at a given nominal setpoint are necessarily identical, and a literal standard deviation of the replicates would be ±0 °C. To avoid the misleading impression of high precision, all MB 1.0 and Kofler hot bench values in Table 2 are instead reported with an estimated resolution-limited uncertainty of ±5 °C, corresponding to one temperature step in this dataset. This value is an instrumental resolution limit, not a statistical standard deviation.

**Figure 1:**
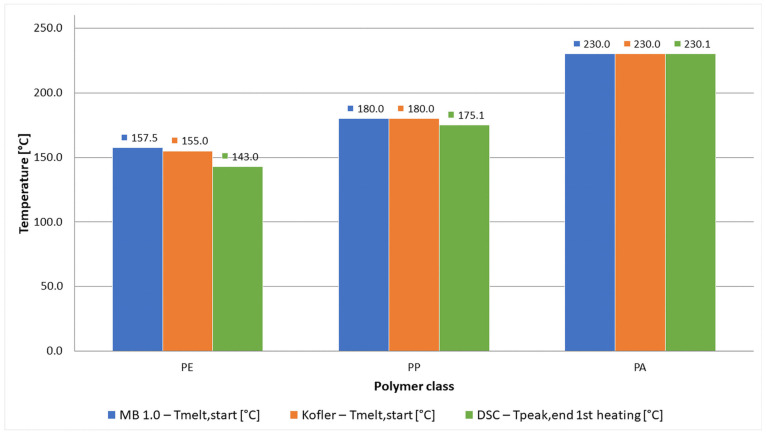
Mean melting temperatures of PE, PP, and PA by method. MB 1.0 and Kofler hot bench bars show mean T_melt,start_; DSC bars show mean first-heating peak-end temperature. The PA/MB 1.0 and PA/Kofler bars are plotted at 230 °C to indicate that no melting was observed at the device’s 230 °C working-range ceiling for the single tested PA sample (PA KGU virgin; *n* = 1), not a measured melting point. Underlying data and calculation are provided in Table S1 (Supplementary Information).

For PE materials, MB 1.0 and the Kofler hot bench record melting ranges of approximately 145–170 °C. DSC first-heating peak-end temperatures for the same four PE samples range from 142.4 to 145.9 °C. The offset between MB 1.0 (T_melt,end_) and DSC (first-heating peak-end) is +20.6 °C on average (range +14.1 to +27.6 °C; *n* = 4), reflecting the substantially higher temperature at which the field-screening device visually registers complete melting compared with the calorimetrically defined peak-end of the same first-heating state. As no manufacturer datasheets were available for this field-sourced net and rope material, the exact PE density grade (e.g., HDPE vs. LLDPE) was not independently verified; the observed DSC range is consistent with HDPE-type material, which is the PE grade predominantly documented in Vietnamese small-scale fishing net production, rather than UHMWPE, a material type not encountered in this fishery context. PP-based blends show consistently higher transition temperatures, namely, T_soft,start_ = (175 ± 5) °C, T_melt,start_ = (180 ± 5) °C, and T_melt,end_ = (200 ± 5) °C, where the ± value denotes the resolution-limited uncertainty of one temperature step rather than a replicate standard deviation (Figure 1).

No complete melting of the PA sample was observed within the MB 1.0 working range (≤230 °C). The DSC first-heating peak-end temperature of the PA KGU virgin reference was 230.1 ± 0.5 °C—measured in the same first-heating thermal state assessed by MB 1.0 and the Kofler hot bench (see Section 2.2)—and lies just above the MB 1.0 working range. This value is close to the typical range for PA-6 in the literature (approx. 215–225 °C) and below that reported for PA-66 (approx. 255–265 °C); first-heating peak-end temperatures are, however, inherently higher than the peak temperatures conventionally reported in the literature, so this comparison remains indicative rather than conclusive. As no manufacturer datasheet was available for this field-sourced net material, we cannot exclude that it is a PA-6/PA-66 copolymer or blend, a technical-grade PA-66 with compounding additives that depress the melting point, or a PA-6 grade with an elevated peak-end value due to processing or weathering history. Confirming the exact polyamide type would require complementary analysis (e.g., FTIR) beyond the scope of this qualitative screening study; samples are therefore designated “PA” (unspecified polyamide type) throughout rather than assigning a specific subtype. The material ranking (PE < PE/PP < PP < PA) is correctly reproduced by both visual methods, and this ranking does not depend on resolving the exact polyamide subtype. The full temperature-resolved response for all samples is shown in Figure S11 (Supplementary Information).

## 4. Discussion

### 4.1. Reproducibility and Method Agreement

The reproducibility of MB 1.0 lies within one temperature step (±5–10 °C), corresponding to the inherent resolution limit of the discrete scale. Agreement with the Kofler hot bench confirms that the handheld device correctly reflects relative differences in melting behavior. For the intended use case—distinguishing polymer classes rather than determining absolute temperature values—this resolution is sufficient.

### 4.2. Comparison with DSC and Published Melting Data

The difference between DSC peak temperature and optically determined T_melt,start_ can be attributed to (1) differing definitions of melting onset, (2) discrete temperature step size of 5–10 K, and (3) temperature gradients between sensor position and sample surface. For PE/PP ropes, DSC resolves clearly separated melting peaks of the PE and PP phases [3,20]; visual methods record the overlapping melting envelope (onset ca. 160 °C; completion up to 190 °C). The full temperature-resolved response for all samples is shown in Figure S11 (Supplementary Information).

### 4.3. Quantitative Assessment of Method Agreement

Given the small number of evaluable sample pairs (*n* = 4–9, depending on parameter) and the discrete, step-limited nature of the MB 1.0 and Kofler measurements, formal parametric significance tests (e.g., t-test or ANOVA) were not applied; such tests would have limited statistical power and their underlying normality assumptions are not well suited to step-quantized data. Method agreement is instead characterized through mean bias, standard deviation of the bias, and range, following common practice for method-comparison studies of this type. For the seven evaluable sample pairs (T_melt,start_), the mean bias is ΔT = +2.9 °C (SD = 9.1 °C; range −10 to +15 °C). For T_soft,start_: bias = +2.5 °C (SD = 2.9 °C; *n* = 4); for T_melt,end_: bias = 0.0 °C (*n* = 6). In the extended dataset (*n* = 9 PE pairs), the mean absolute deviation between MB 1.0 and Kofler is 2.8 °C. For PE samples, the mean offset T_melt,end_ (MB 1.0) vs. DSC first-heating peak-end is +20.6 °C (range +14.1 to +27.6 °C; *n* = 4).

Polymer class separation based on T_melt,start_ (MB 1.0): PE vs. PP ≥ +20 °C; PP vs. PA > +50 °C. All separation distances exceed the instrumental uncertainty (±10 °C) by at least a factor of two.

### 4.4. Contextualization Within the Analytical Method Spectrum

Table 3 provides an overview of polymer identification methods, compared on practical deployment characteristics (cost, field-deployability, calibration requirements, resolving power, and applicability to dark or contaminated samples) rather than on the underlying detection principle. The methods listed differ fundamentally in what they measure: DSC, the Kofler hot bench, and MB 1.0 detect thermal transitions (melting behavior), whereas NIR and ATR-FTIR identify chemical bonds via spectroscopic absorption. MB 1.0 occupies a position between the Kofler hot bench and density separation, combining field deployability with temperature-based polymer discrimination applicable to blended and contaminated samples. Its cost of approximately 150–200 EUR is more than one order of magnitude below the Kofler hot bench (ca. 1820 EUR [21]) and two to three orders of magnitude below DSC systems.

### 4.5. Relevance for Resource-Constrained Contexts

Vietnam is among the major contributors to ocean plastic pollution globally [22], and Southeast Asian countries generate large quantities of inadequately managed plastic waste annually [23]. Plastic sorting is carried out predominantly by informal workers without access to analytical instruments [24]. MB 1.0 can provide the polymer discrimination required for pre-sorting operations (PE, PP, and PA) under these conditions.

### 4.6. Limitations

The subjectivity of visual assessment is a principal limitation; inter-operator variability was not systematically investigated. Standardized training materials with photographic references—provided as Figures S2–S10 (Supplementary Information)—can reduce inter-operator variability, but systematic inter-operator studies were not conducted. The temperature calibration of MB 1.0 is not traceable; a procedure using low-melting reference substances (e.g., tin, T_melt_ = 232 °C) could improve measurement quality. The temperature range is limited to approximately 230 °C; the polyamide reference (DSC first-heating peak-end: 230.1 °C) cannot be fully characterized, and its exact subtype (PA-6, PA-66, or copolymer) could not be independently verified in the absence of a manufacturer datasheet (see Section Transition Temperatures and Comparison with DSC). Sample mass and geometry were not individually recorded; masses were kept below 0.5 g to ensure rapid thermal equilibrium and minimize thermal gradients within the specimen, but this limits quantitative correlation between mass/geometry and observed transition temperatures. Used fishing net and rope samples may additionally carry fishery-related surface contamination (biofouling, salt residues, and oils) alongside manufacturing additives (dyes and stabilizers), either of which could shift observed transition temperatures; this was not separately assessed. MB 1.0 is aimed almost exclusively at sorting fishing net and rope waste, involving polymer classes with substantially different melting points (typically > 30–50 °C apart, e.g., PE, PP, and PA). Within this application it performs well and is highly cost-effective. It is not capable of efficiently distinguishing polymers with melting-point differences below approximately 20 °C (e.g., closely spaced PE density grades), nor of assessing polymers with melting points above its 230 °C working range.

## 5. Conclusions

This study validates MB 1.0, a low-cost (150–200 EUR), field-deployable device for qualitative melting-range screening of fishing-net-derived thermoplastics. Across the tested materials, MB 1.0 reliably distinguishes PE, PE/PP blends, PP, and PA, reproducing the material ranking (PE < PE/PP < PP < PA) confirmed independently by the Kofler hot bench, DSC, and values in the published literature [25,26,27].

Quantitative comparison against the Kofler hot bench shows good agreement (mean bias for T_melt,start_: +2.9 °C; SD = 9.1 °C; *n* = 7). A systematic offset of +20.6 °C (range +14.1 to +27.6 °C) was observed against DSC first-heating peak-end temperatures for PE, reproducible across the extended dataset (mean absolute deviation 2.8 °C; *n* = 9 PE pairs; DSC reproducibility CV = 0.64%). This offset is consistent and predictable, so the device’s qualitative classification is not compromised despite the absence of instrument-level calibration.

MB 1.0 does not replace quantitative thermal analysis: it cannot resolve absolute melting temperatures, reliably separate polymers with closely spaced melting points, or independently confirm polyamide subtype. Calibration traceability, inter-operator reproducibility, and the influence of fishery-related sample contamination were identified as open limitations (Section 4.6).

Given its low cost and field portability, MB 1.0 is intended as a pre-sorting screening complement to laboratory-grade analytics in resource-constrained recycling contexts such as small-scale Vietnamese fishing net recovery, where DSC and NIR instrumentation are typically unavailable [22,23,24]. Field validation, a traceable calibration protocol, and a systematic inter-operator study are identified as priorities for future work.

## Figures and Tables

**Table 1:** Sample overview.

Designation	Material/Application	Origin/Remarks	Method(s)
PE B new	PE, virgin grade B	Lab sample, Vietnam	MB, Kofler, DSC
PE HLU Virgin net	PE, virgin net material	Lab sample, Vietnam	MB, Kofler, DSC
PE A new Granulat	PE, virgin grade A	Vietnam	MB, Kofler, DSC
PE A new net	PE, virgin net	Vietnam	DSC only
PE Lupolen	PE, Lupolen type	Datasheet available	MB, Kofler, DSC
NTU virgin net	PE, virgin net (NTU)	Vietnam	MB, DSC
NTU roof 2-month net	PE, weathered net (2 months outdoors)	Vietnam	MB, Kofler
PE/PP dark Ropes specimen	PE/PP blend (injection-molded)	Vietnam	MB, Kofler, DSC
PE/PP Rope	PE/PP blend, rope	Vietnam	MB, Kofler, DSC
PE/PP bright Rope (0–5× washed)	PE/PP blend, rope, washing series	Vietnam	DSC only
PP Mixture blue	PP blend, flakes, blue	Sorting facility Vietnam	MB, Kofler, DSC
PP Mixture green	PP blend, flakes, green	Sorting facility Vietnam	MB, Kofler, DSC
PA KGU virgin	PA, virgin (type not independently verified)	Reference material	MB, Kofler, DSC

**Table 2:** Transition temperatures for selected samples (all values in °C). For MB 1.0 and the Kofler hot bench, the reported ± value is the resolution-limited uncertainty of one discrete temperature step (5 °C in this dataset), not a statistical standard deviation of the replicates. For DSC, the reported value is the standard deviation of triplicate measurements.

Sample	Polymer	Method	T_soft,start_ [°C]	T_melt,start_ [°C]	T_melt,end_ [°C]	T_peak,end_ DSC First Heating [°C]	T_peak_ DSC Second Heating [°C]
PE B new	PE	MB 1.0	145 ± 5	160 ± 5	160 ± 5	–	
PE B new	PE	Kofler	140 ± 5	145 ± 5	160 ± 5	145.9 ± 0.9 (PE)	136.5 ± 1.3 (PE)
PE HLU Virgin net	PE	MB 1.0	145 ± 5	160 ± 5	170 ± 5	–	–
PE HLU Virgin net	PE	Kofler	140 ± 5	145 ± 5	170 ± 5	142.4 ± 0.5 (PE)	133.7 ± 0.3 (PE)
PE A new Granulat	PE	MB 1.0	140 ± 5	145 ± 5	160 ± 5	–	–
PE A new Granulat	PE	Kofler	145 ± 5	150 ± 5	160 ± 5	144.0 ± 0.7 (PE)	136.4 ± 1.1 (PE)
PE Lupolen	PE	MB 1.0	145 ± 5	160 ± 5	170 ± 5	–	–
PE Lupolen	PE	Kofler	–	170 ± 5	≥190	145.3 ± 0.4 (PE)	137.4 ± 1.2 (PE)
PE/PP dark Ropes	PE/PP	MB 1.0	–	160 ± 5	180 ± 5	–	–
PE/PP dark Ropes	PE/PP	Kofler	–	160 ± 5	180 ± 5	133.7 ± 0.8 (PE) 179.3 ± 1.0 (PP)	125.0 ± 0.4 (PE) 169.5 ± 0.3 (PP)
PE/PP Rope	PE/PP	MB 1.0	–	160 ± 5	190 ± 5	–	–
PE/PP Rope	PE/PP	Kofler	–	160 ± 5	190 ± 5	146.5 ± 2.3 (PE) 177.6 ± 2.5 (PP)	137.3 ± 0.8 (PE) 168.5 ± 0.1 (PP)
PP Mixture blue	PP	MB 1.0	175 ± 5	180 ± 5	200 ± 5	–	–
PP Mixture blue	PP	Kofler	175 ± 5	180 ± 5	200 ± 5	172.7 ± 0.8 (PP)	163.0 ± 1.3 (PP)
PP Mixture green	PP	MB 1.0	175 ± 5	180 ± 5	200 ± 5	–	–
PP Mixture green	PP	Kofler	175 ± 5	180 ± 5	200 ± 5	170.9 ± 0.9 (PP)	165.9 ± 1.1 (PP)
PA KGU virgin	PA	MB 1.0	–	–	No reaction up to 230 °C	–	–
PA KGU virgin	PA	Kofler	–	–	No reaction up to 230 °C	230.1 ± 0.5 (PA)	221.8 ± 0.2 (PA)

**Table 3:** Comparison of polymer identification methods for fishing net recyclates.

Method	Cost (Approx.)	Field-Deployable	Calibration	Polymer Resolution	Dark/Contam. Samples
DSC	10,000–60,000 EUR	No	Extensive	Quantitative	Yes
ATR-FTIR (stationary)	15,000–40,000 EUR	No	Moderate	Complete	Limited
Portable NIR (handheld)	ca. 15,000 EUR	Conditional	Low	Complete	No
Kofler hot bench	ca. 1820 EUR	No	Moderate	Qualitative	Yes
MB 1.0	ca. 150–200 EUR	Yes	Low	Qualitative	Yes
Density separation	<10 EUR	Yes	None	Coarse	Yes

## Data Availability

The original contributions presented in this study are included in the article/Supplementary Material. Further inquiries can be directed to the corresponding author.

## Supplementary Materials

The following supporting information can be downloaded at: https://www.mdpi.com/article/10.3390/polym18151809/s1, Figure S1: Schematic layout of MB 1.0 with component labels; Figures S2–S10: Illustrated step-by-step operating procedure; Figure S11: Color-coded temperature matrix of all measurement data; Table S1: Mean melting temperatures underlying Figure 1, by polymer class and method.
