# Supplementary material for: Low-Cost Qualitative Screening of Melting Ranges in Recycled Fishing Net Plastics: Comparison of Portable Handheld Device with Kofler Hot Bench and DSC
Source: Polymers (Basel). 2026 Jul 24;18(15):1809. doi: 10.3390/polym18151809 (PMC13467709; doi:10.3390/polym18151809)
Supplement: Supplementary file 1 [file polymers-18-01809-s001.zip › polymers-4426715-supplementary.pdf]

Supporting Information

Low-Cost Qualitative Screening of Melting Ranges in Recycled Fishing Net Plastics: Comparison of a Portable Handheld Device with Kofler Hot Bench and DSC

Sylvia Waschke, Julia Tetzner, Thomas Potempa, Max Ehleben

Institute of Recycling (IFR), Ostfalia University of Applied Sciences, Wolfsburg, Germany

* Correspondence: potempa@ostfalia.de

Contents

S1. Device Schematic of the Handheld Melting Range Instrument (Melting Box 1.0)

S2. Film Sequences: Melting Box 1.0 — Illustrated Operating Procedure

S3. Overview of All Measurement Data (Colour-coded Temperature Matrix)

S4. Melting Range Data Underlying Figure 1 (Table S1)

S1. Device Schematic of the Handheld Melting Range Instrument (Melting Box 1.0)

Figure S1 shows the schematic layout of the Melting Box 1.0. The device integrates the essential components for controlled heating of small plastic samples into a compact, portable unit.

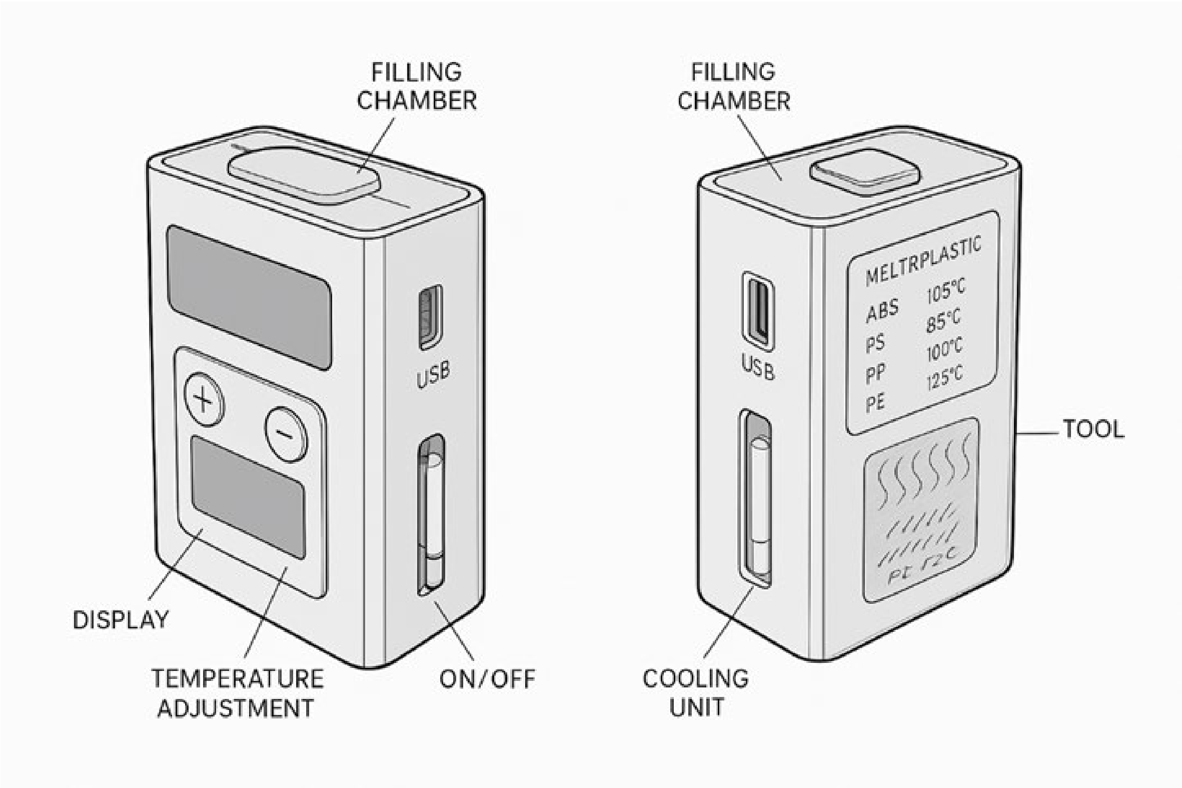

Figure S1. Schematic layout of the handheld melting range instrument (Melting Box 1.0, based on the Flowermate V5.0s PRO Vaporizer [27]). 1 = Heating element (electrically heated ceramic block); 2 = Sample contact surface (flat-ground ceramic surface); 3 = Temperature sensor (integrated sensor of the electronic control unit); 4 = Housing / thermal insulation; 5 = Control unit with digital OLED display and temperature adjustment buttons (+/−); 6 = Power supply (lithium-ion battery, 2600 mAh, with AC/DC adapter).

S2. Film Sequences: Melting Box 1.0 — Illustrated Operating Procedure

The following image sequence illustrates the standard procedure for the qualitative melting range determination of plastic samples using the Melting Box 1.0. Sequences 1–10 correspond to the steps shown in the accompanying video.

Sequence 1: Introduction & Overview

Device and accessories are laid out: Melting Box 1.0, aluminium stock, scissors, plunger rod, crucible, and a piece of fishing net material.

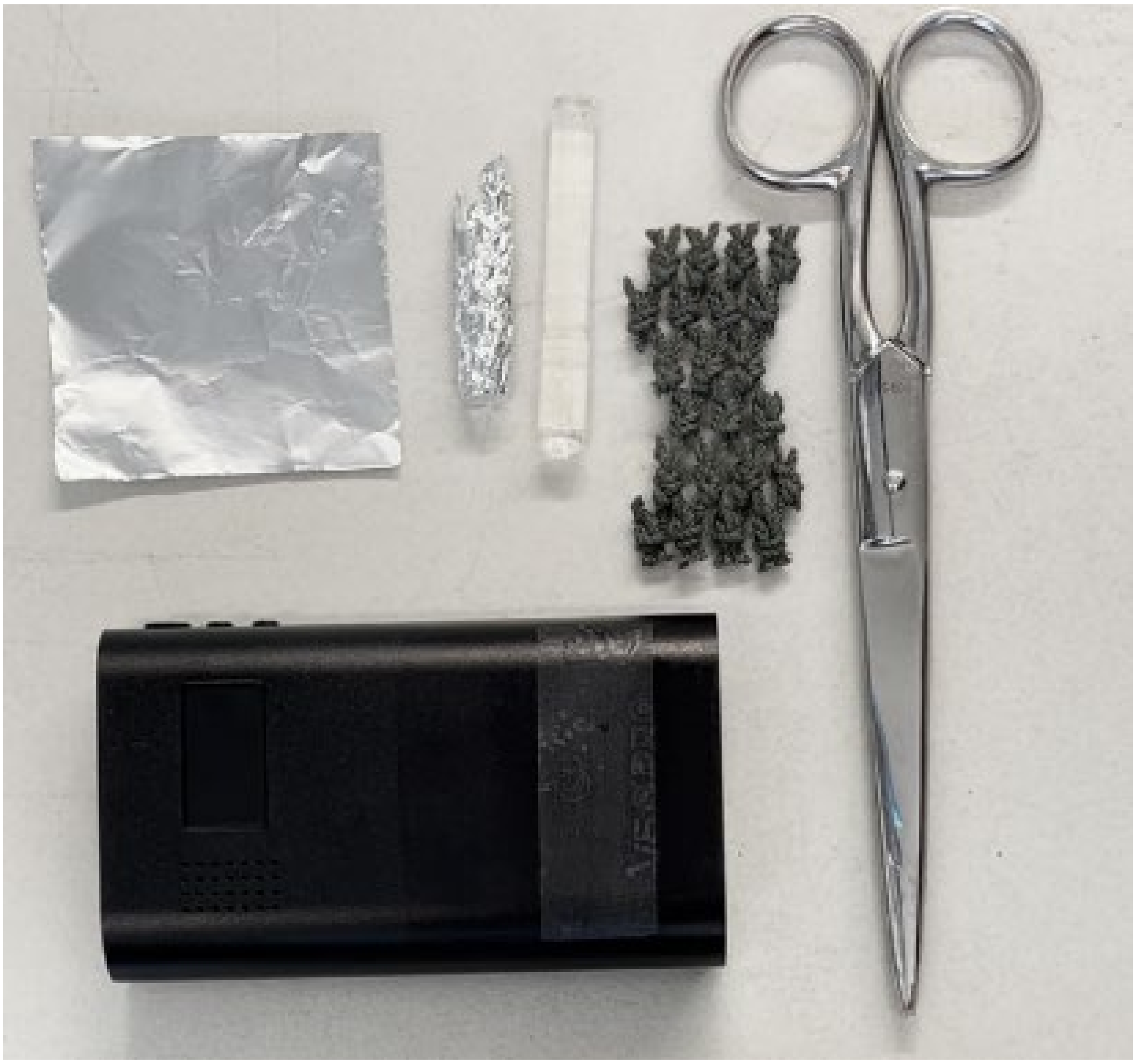

Figure S2. Sequence 1: Overview of the device and needed accessories.

Sequence 2: Device Components

Details of the Melting Box 1.0: display, heating chamber, outlet; insertion and removal of the plunger rod.

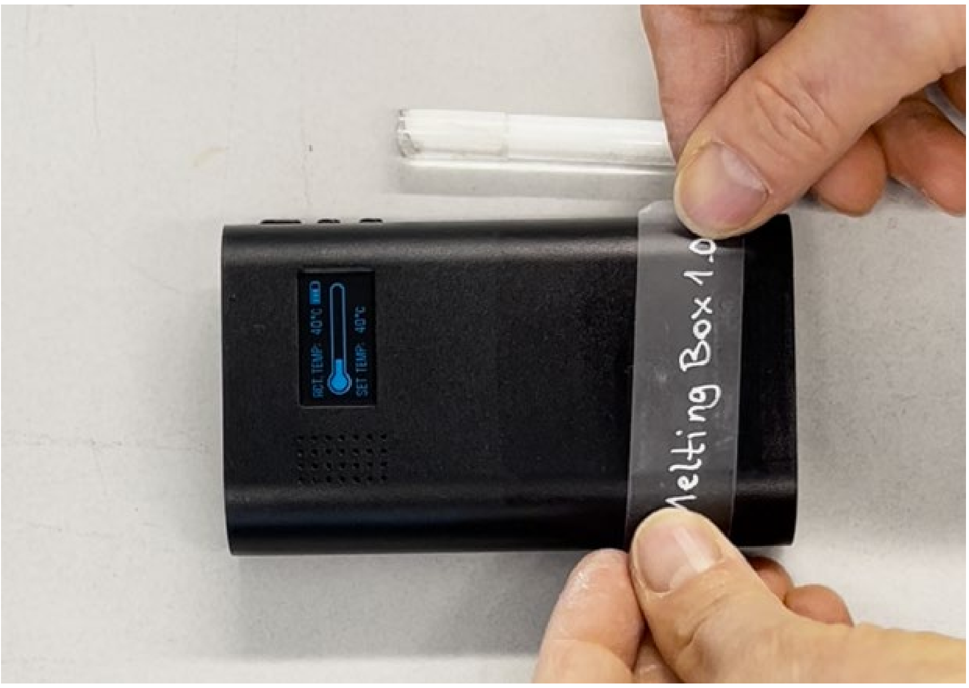

Figure S3. Sequence 2: Components of the Melting Box 1.0 (display, heating chamber, plunger rod).

Sequence 3: Preparation of the Aluminium Crucible

An aluminium crucible is formed from a piece of aluminium stock and prepared for sample loading.

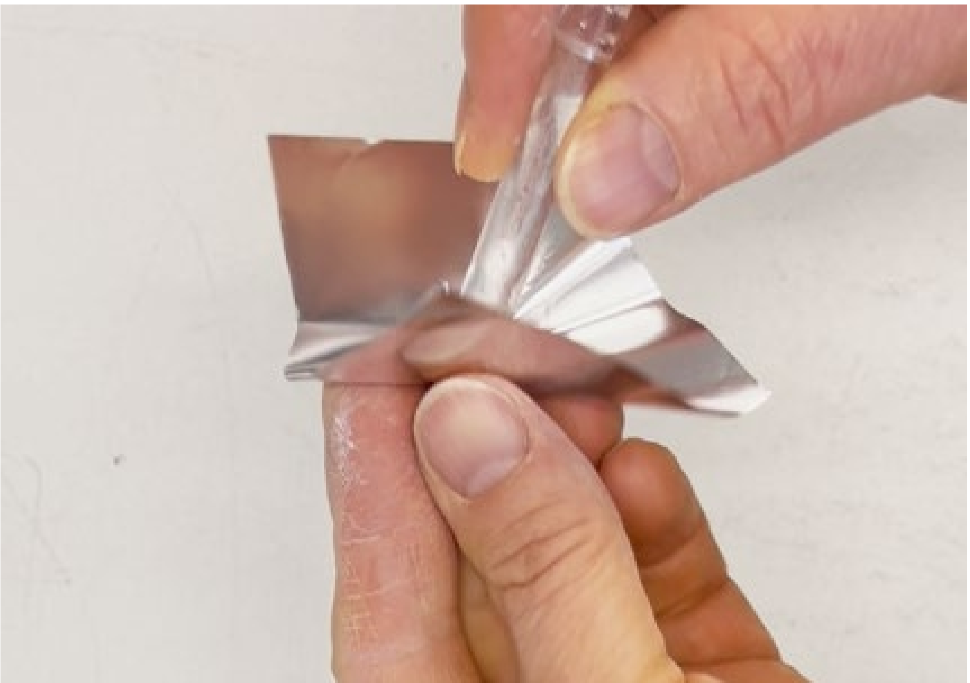

Figure S4. Sequence 3: Shaping the aluminium crucible.

Sequence 4: Preparation of the Fishing Net Recyclate

A piece of fishing net is cut and placed into the aluminium crucible.

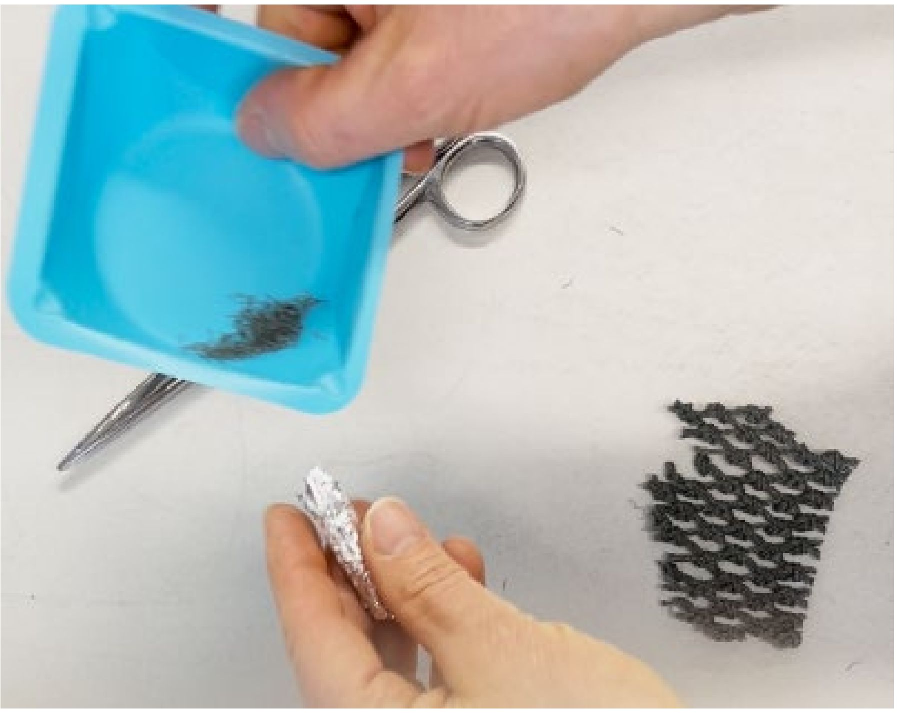

Figure S5. Sequence 4: Loading the fishing net material sample into the aluminium crucible.

Sequence 5: Heating the Device

The target temperature is set on the device. The temperature display shows the heat-up progress; a 30-second pre-heating phase is observed.

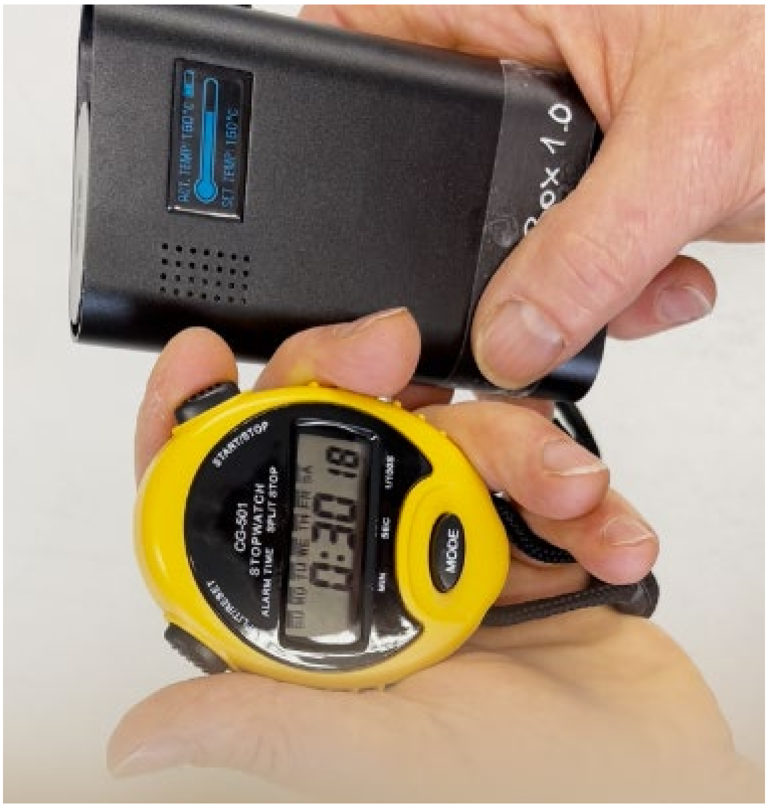

Figure S6. Sequence 5: Temperature setting and heating phase (30 s pre-heating period).

Sequence 6: Force Calibration (Laboratory Balance)

The Melting Box 1.0 is loaded with crucible and plunger rod. The laboratory balance is tared. The operator slowly presses down the plunger rod; the balance display shows the applied force (1000–2000 g).

Caption/Voiceover: *"Before use, the force to be applied is set and verified on the laboratory balance. This ensures a precise and reproducible process."*

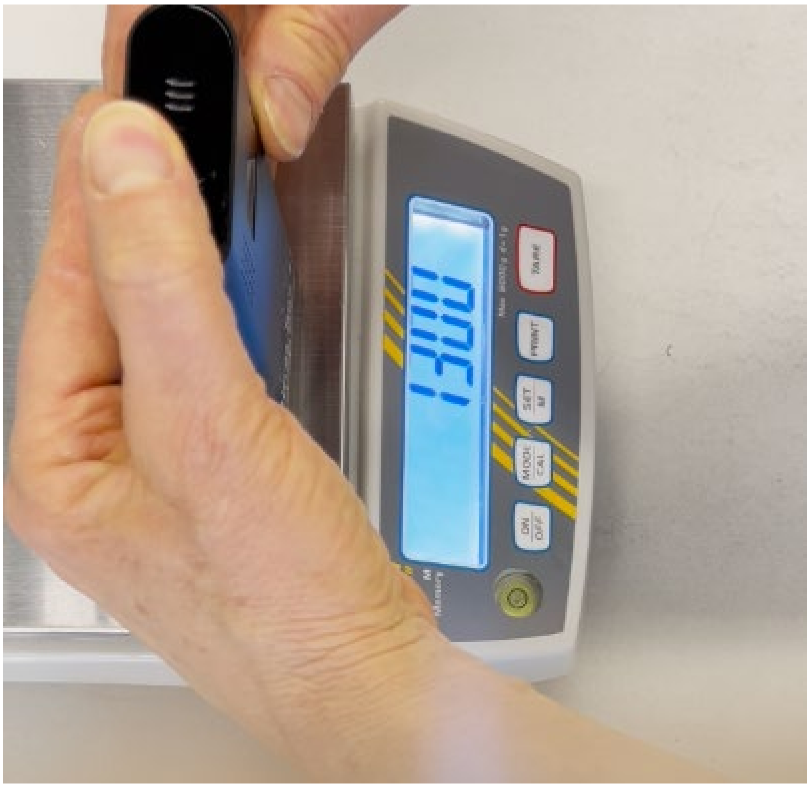

Figure S7. Sequence 6: Calibration of the contact force using a laboratory balance (target range: 1000–2000 g).

Sequence 7: Melting Process

The preheated device is loaded with the filled crucible. The plunger rod is pressed with the calibrated force. Temperature and timer run; the material melts and is compressed.

Caption/Voiceover: *"During the actual melting step, the previously calibrated force is applied."*

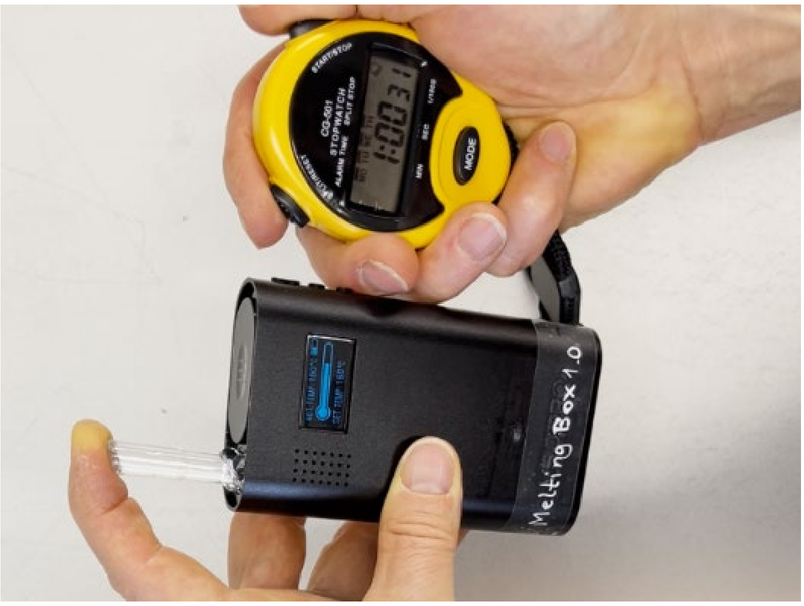

Figure S8. Sequence 7: Melting process with controlled force application.

Sequence 8: Process Observation

Close-up of the melting process: visual assessment of the material behaviour (softening, flow, droplet formation) according to the 0/*/x rating scale.

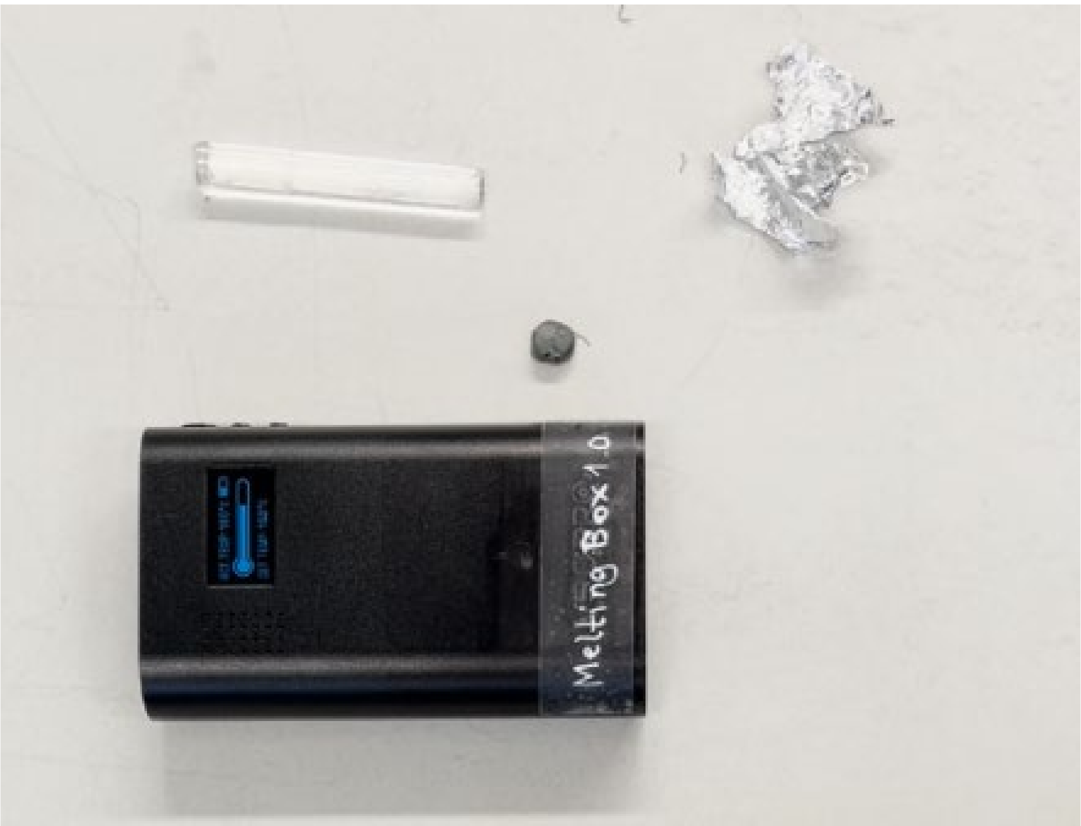

Figure S9. Sequence 8: Visual assessment of melting behaviour according to the 0/*/x rating scale.

Sequence 9: Sample Retrieval & Result

The device is opened. Crucible and plunger rod are removed. The resulting plastic disc is retrieved and presented.

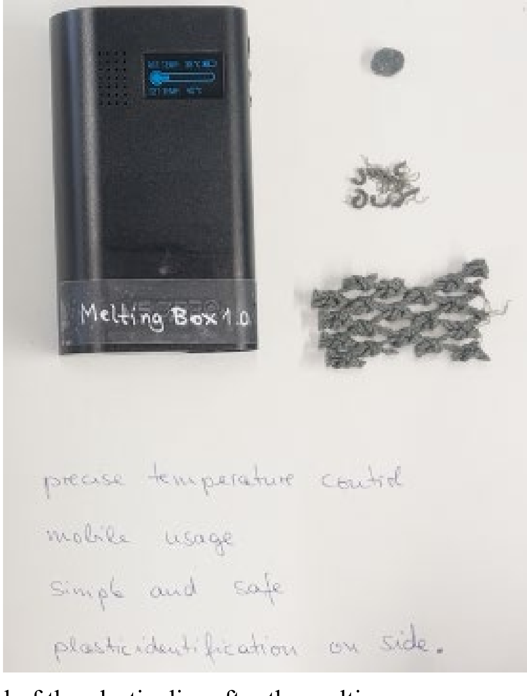

Figure S10. Sequence 9: Retrieval of the plastic disc after the melting process.

Sequence 10: Summary & Advantages

Device and product are presented side by side. Key advantages of the Melting Box 1.0:

- Portable and field-deployable
- Low device cost (approx. 150–200 EUR)
- Simple operation without laboratory infrastructure
- Qualitative discrimination of PE, PP, and PA is feasible
- Reproducible results through calibrated force application

S3. Overview of All Measurement Data (Colour-coded Temperature Matrix)

Figure S11 shows a colour-coded overview of all measurement data from MB 1.0, Kofler hot bench, and DSC for all samples examined in this study. Each cell represents the temperature recorded at a given measurement step for a given sample. Colour coding: yellow = no visible reaction (0); orange = softening (*); red = pronounced melting (x). DSC peak temperatures (T_{peak}) are indicated by vertical reference cells. The temperature axis runs from 130 °C to 230 °C

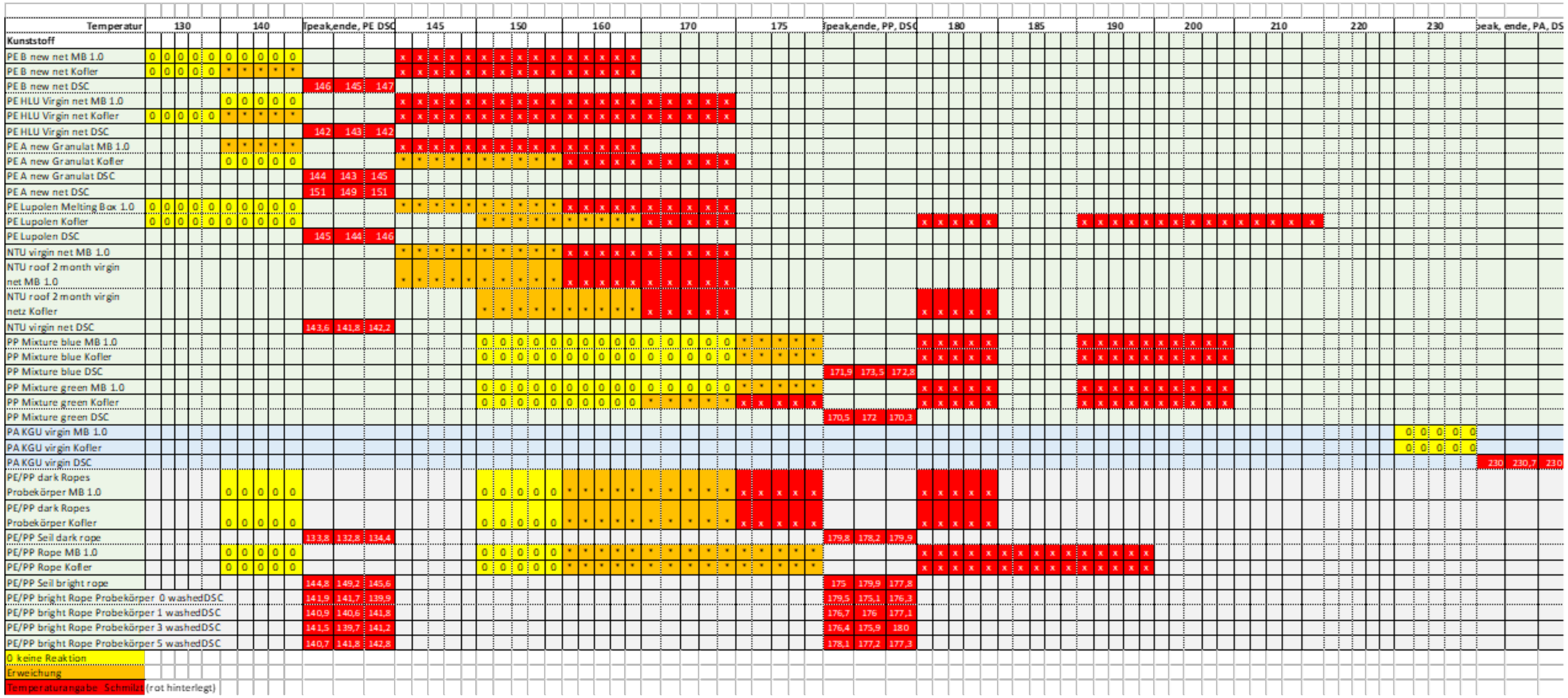

Figure S11. Colour-coded temperature matrix of all melting range measurements. Rows: individual samples with the respective measurement method (MB 1.0 = Melting Box 1.0, Kofler = Kofler hot bench, DSC = differential scanning calorimetry). Columns: temperature steps from 130 °C to 230 °C. Replicate measurements (n = 5) are shown individually for MB 1.0 and Kofler.

S4. Melting Range Data Underlying Figure 1 (Table S1)

Table S1 lists the mean values underlying Figure 1 (main text), computed from the per-sample raw data in Table 2 (main text). MB 1.0 and Kofler hot bench values are mean Tmelt,start; DSC values are mean Tpeak. n denotes the number of samples averaged for each cell.

Table S1. Mean melting temperatures by polymer class and method, underlying Figure 1 (main text).

Polymer class	MB 1.0 – mean Tmelt,start [°C]	n	Kofler – mean Tmelt,start [°C]	n	DSC – mean Tpeak [°C]	n
PE	157.5	6	155.0	6	143.0	6
PP	180.0	2	180.0	2	175.1	4
PA	230.0[a]	1	230.0[a]	1	230.1	1

[a] No melting was observed for the PA sample (PA KGU virgin) at the 230 °C ceiling of either the MB 1.0 or the Kofler hot bench (n = 1 each); 230 °C is plotted as a right-censored marker in Figure 1, not a measured melting point. See Table 2 (main text) for the full raw data underlying this table.
